# Copper ions, prion protein and Aβ modulate Ca levels in central nervous system myelin in an NMDA receptor-dependent manner

**DOI:** 10.1186/s13041-022-00955-2

**Published:** 2022-07-26

**Authors:** Shigeki Tsutsui, Megan Morgan, Hugo Tedford, Haitao You, Gerald W. Zamponi, Peter K. Stys

**Affiliations:** 1 Department of Clinical Neurosciences, University of Calgary, Calgary, AB T2N 4N1 Canada; 2 Department of Physiology and Pharmacology, University of Calgary, Calgary, AB T2N 4N1 Canada; 3 Hotchkiss Brain Institute, University of Calgary, Calgary, AB T2N 4N1 Canada; 4 Alberta Children’s Hospital Research Institute, University of Calgary, Calgary, AB T2N 4N1 Canada

**Keywords:** Alzheimer's disease, Bathocuproine, Proteolipid protein, Glutamate receptor

## Abstract

As in neurons, CNS myelin expresses N-Methyl-D-Aspartate Receptors (NMDARs) that subserve physiological roles, but have the potential to induce injury to this vital element. Using 2-photon imaging of myelinic Ca in live ex vivo mouse optic nerves, we show that Cu ions potently modulate Ca levels in an NMDAR-dependent manner. Chelating Cu in the perfusate induced a substantial increase in Ca levels, and also caused significant axo-myelinic injury. Myelinic NMDARs are shown to be regulated by cellular prion protein; only in prion protein KO optic nerves does application of NMDA + D-serine induce a large Ca increase, consistent with strong desensitization of these receptors in the presence of prion protein limiting Ca overload. Aβ_1-42_ peptide induced a large Ca increase that was also Cu-dependent, and was blocked by NMDAR antagonism. Our results indicate that like in neurons, myelinic NMDARs permeate potentially injurious amounts of Ca, and are also potently regulated by micromolar Cu and activated by Aβ_1-42_ peptides. These findings shed mechanistic light on the important primary white matter injury frequently observed in Alzheimer's brain.

**Supplementary Information:**

The online version contains supplementary material available at 10.1186/s13041-022-00955-2.

NMDARs in neurons are potently modulated by a complex interplay between copper and cellular prion protein (PrP^c^). We showed that absence of prion protein leads to increased NMDAR activity due to alterations of co-agonist sensitivity, and that chelating copper by either BCS or Aβ had a similar effect, leading us to conclude that copper ions mediate their regulatory action via PrP^c^ [1]. Traditionally the myelin sheath was considered an inert lipid-rich insulating layer that does not participate in signaling, and is not subject modification other than in disease states. Both oligodendrocytes and the myelin sheath itself express functional ionotropic glutamate receptors [2], and we reported chemical neurotransmission from axon to myelin via a novel axo-myelinic synapse [3]. This suggests that myelin is a dynamic signaling partner that can undergo physiological modulation, and importantly, could be a direct target for a variety of acute and chronic disorders that affect white matter, including pathology seen in Alzheimer's disease. Here we show, that like in neurons, copper ions operating via PrP^c^ profoundly modulate the response of myelin to NMDAR activation. We also show how Aβ signals via this mechanism, leading to myelin Ca overload, suggesting that this peptide or its toxic oligomers could directly induce white matter pathology in Alzheimer’s disease.

Wild-type C57 mice were purchased from Charles River and PrP^C^ knockout (KO) mice were kindly provided by Dr. Frank Jirik. Optic nerves were dissected free, maintained in a heated perfusion chamber and imaged using 2-photon microscopy as previously described [4]. Myelin was stained with the green lipophilic dye DiOC_6_(3) and co-localized red signal from the Ca reporter XRhod1 reflected Ca levels in the cytosolic compartments of myelin (Fig. 1). With physiological concentrations of Cu and glycine (1 µM each) in the perfusate, application of the non-transportable NMDAR co-agonist D-serine (10 µM) failed to increase Ca in wild type mouse optic nerve myelin, nor did addition of NMDA (500 µM). In contrast, chelating Cu induced a substantial but reversible increase in myelinic Ca. This increase was completely blocked by the NMDAR antagonist DCKA (50 µM), as well as by Cu added in molar excess of BCS. Moreover, BCS induced significant axo-myelinic injury in an NMDAR-dependent manner (Additional file 1). More selective block of GluN2A and 2B-containing NMDARs with ifenprodil (9 µM) + NVP (1 µM) was ineffective. These results indicate that in the presence of physiological Cu levels, exogenous NMDAR agonists failed to promote myelinic Ca accumulation, but Cu removal alone induced a substantial Ca increase that was mediated by non-GluN2A/2B-containing NMDARs. We then investigated how Cu ions could exert their effect on NMDAR function. PrP^C^ is one of the most important Cu-binding proteins in the CNS [5]. In striking contrast to WT mice, PrP KO optic nerve myelin exhibited a substantial Ca increase in response to D-serine and NMDA., which was completely blocked by DCKA (Fig. 1D). These data are consistent with our previous findings in neurons showing that PrP^C^ regulates co-agonist affinity/sensitivity [6].

**Fig. 1:**
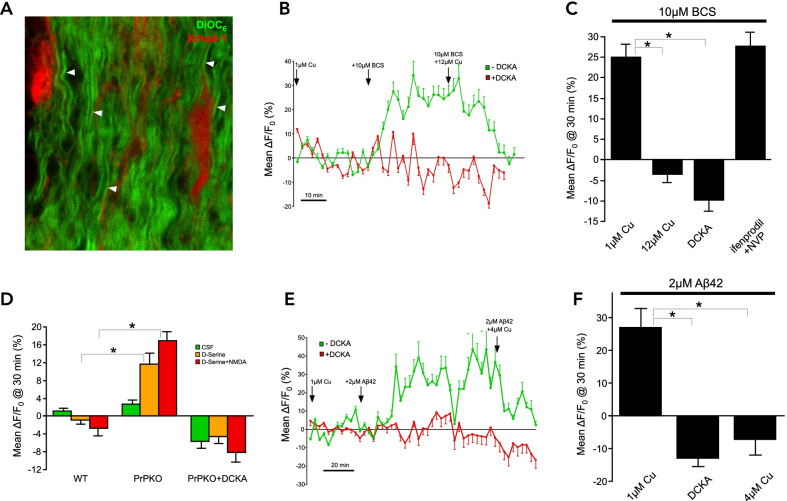
**A** Example 2-photon micrograph of mouse optic nerve whose myelin was labeled with the green lipophilic dye DiOC6 together with the Ca indicator Xrhod-1. Individual myelin sheaths are readily visible (arrows). **B** Myelinic Ca levels increased substantially by removal of free Cu ions with BCS. This effect was blocked by the broad spectrum NMDAR antagonist DCKA (50 µM). **C** Quantitative result showing how excess Cu or NMDAR blockade (DCKA) prevented BCS-induced Ca increase. Selective antagonism with GluN2A & B-containing NMDARs with ifenprodil and NVP-AAM077 was not effective. **D** Activating NMDARs with D-serine and NMDA failed to induce a Ca rise in WT optic nerve. In contrast, PrP-lacking myelin exhibited a substantial Ca increase in response to a glycine site agonist alone (D-serine, 10 µM), which increased further with addition of NMDA (500 µM). These responses were completely blocked by DCKA confirming the major contribution of NMDARs. **E**, **F** Aβ1-42 also caused a substantial myelinic Ca increase which could be overcome by excess Cu ions or NMDAR antagonists. Taken together, these data indicate that myelinic NMDARs can admit substantial amounts of Ca, and their activity is potently modulated by micromolar Cu and prion protein. The effect of exogenous Aβ_1–42_ has implications for white matter pathology frequently seen in Alzheimer's disease (*P < 0.01, Dunn's Many-to-One Rank Comparison Test)

Aβ peptides are thought to play a central role in the pathogenesis of Alzheimer’s disease [7]. Aβ monomers are also potent chelators of Cu [8], and in their oligomeric form, have been shown to bind with PrP^C^ [9], potentially altering the physiological role of this protein. Moreover, white matter pathology is a prominent feature of Alzheimer’s disease [10], and given the important role of both Cu and PrP^C^ in regulating myelinic Ca levels, we therefore tested the effects of exogenously applied synthetic Aβ_1–42_ peptides on Ca responses in optic nerve myelin. Application of 2 µM Aβ_1–42_ (in the presence of 1 µM Cu) significantly increased myelinic Ca to levels similar to those observed with BCS. The effect was reversible within 10–20 min after discontinuation of Aβ. As with BCS, the Aβ-mediated myelinic Ca increase was completely blocked by NMDAR antagonism with DCKA or by an excess of added Cu ions (Fig. 1E, F). Myelin is injured in 5xFAD Alzheimer’s mice suggesting Aβ can reach toxic levels in vivo as well (Additional file 2).

Glutamate is the main excitatory neurotransmitter in the mammalian CNS, essential for synaptic transmission subserving key functions such as learning and memory [11]. Chemical synaptic transmission has traditionally been ascribed to neurons, but recent data indicate that neurons can signal glial cells (astrocytes, oligodendrocytes and their precursors) using conventional neurotransmitters such as glutamate and GABA [12]. This has now been extended to myelin itself with emergence of an axo-myelinic synapse, whereby action potential traffic along an axon releases glutamate to activate “post-synaptic” AMPA and NMDA receptors on the adaxonal myelin [13] (Additional file 3). One plausible role could be for the axon to signal its supporting oligodendrocyte, in accordance with the volume of action potential traffic, to produce more lactate for axonal ATP production by internodal mitochondria [14]. Another intriguing possibility is for such a synapse to modulate myelin structure and biochemical composition in response to activity, possibly contributing to learning [15]. On the flip side, such a synapse could play a role in pathophysiological responses of white matter to acute (ischemia, trauma) or chronic (schizophrenia, MS, Alzheimer’s) insults. Here we show that like in neurons [6], myelinic Ca fluctuations in response to NMDAR activation are strongly modulated by Cu ions, with a key role played by endogenous PrP^c^, both likely acting to accelerate desensitization of NMDARs [6] to limit damaging Ca entry into the cytosolic spaces of myelin. In support of chronic and primary degeneration of white matter in Alzheimer’s, we also show that Aβ strongly affects glutamatergic signaling across the axo-myelinic synapse, inducing substantial Ca increases by perturbing the normal modulatory role of Cu ions. Moreover, Alzheimer's white matter exhibits marked alterations in proteolipid protein, the major protein of CNS myelin, which is consistent with this notion (Additional file 4). Our results provide important insight into molecular mechanisms of myelin damage, with relevance to both acute and chronic disorders of the CNS where white matter pathology is prominent.

## Supplementary Information

**Additional file 1. Fig. S1.** Freshly dissected optic nerves from mice expressing green YFP in axons, with myelin labeled using the lipid probe Nile Red, were incubated in oxygenated aCSF at 35°C with 1 μM CuSO_4_ added. Nerves were then fixed in PFA and imaged intact by confocal microscopy. A) After 6 hrs of incubation in aCSF alone, axons and myelin remained morphologically intact. B) In contrast, a 6 hr incubation with addition of the Cu chelator BCS (10 μM) induced significant pathology in the form of axomyelinic spheroids (arrows). C) Quantitative analysis showing mean # of spheroids per 250x250 μm field of view. The BCS-induced pathology was completely blocked by the NMDAR antagonist 5,7-dichlorokynurenic acid (50 μM).

**Additional file 2. Fig. S2.** 9 month old C57 wild type or 5xFAD (transgenic mice harboring 5 human Alzheimer's mutations involving presenilin and APP (Oakley et al., 2006) were immunolabeled for citrullinated myelin basic protein (citMBP, 1B8 antibody) and Aβ (6E10 antibody), and counter-stained with DAPI. Representative micrographs show no amyloid plaque deposition in the wild type mouse as expected and minimal citMBP in the corpus callosum (CC) indicative of healthy myelin. In contrast, the 5xFAD mouse exhibited heavy plaque deposition typically seen at this advanced age, including in the corpus callosum (*). In this white matter tract notable citMBP signal was observed (arrows) consistent with biochemically damaged myelin. These data are consistent with the notion that in vivo Aβ reaches levels sufficient to induce myelin abormalities, which also appears to occur in the human (Additional file 4).

**Additional file 3. Fig. S3.** Schematic diagram of the proposed signaling arrangement of myelinic NMDARs and their regulation by Cu ions and PrP^c^. Action potentials conducted along myelinated axons release glutamate into the periaxonal space (Micu et al, 2016) which, together with the obligatory co-agonist glycine or D-serine, activates myelinic NMDARs resulting in physiological Ca increases in myelin. This is under potent control of Cu ions likely exerting their effects via PrP^c^ ①. Genetic ablation of PrP^c^ increases the sensitivity of NMDARs to agonist leading to increased myelinic Ca entry ②. Acute reduction of Cu levels in the periaxonal space by chelators such as BCS or Aβ_1–42_ impairs the ability of PrP^c^ to regulate NMDARs resulting in excessive receptor activation and Ca-mediated injury to the myelin sheath. Thus, Cu ions in concert with PrP^c^ may be fundamental regulators of physiological glutamatergic signaling across the axo-myelinic synapse; disruption of this mechanism may represent the earliest steps of demyelinating pathology.

**Additional file 4. Fig. S4.** Using standard methods, SDS PAGE of 10% human white matter homogenate from 4 non-neurological control and 3 Alzheimer's disease subjects matched for age. Blots were probed using a proteolipid protein monoclonal antibody (#MA1-80034, Thermofisher). A) In controls, most signal was restricted to the monomeric form, as well as the lower molecular weight DM20 splice variant. In striking contrast, all 3 AD samples exhibited very high molecular weight PLP aggregates that resisted the denaturing conditions of the gel. B) Summary of the densitometry analysis plotted as the integral of very high MW bands > 250 kDa as a ratio of monomer intensity. With the other data presented in the paper, these results are consistent with the notion that excess myelinic Ca accumulation via NMDAR's dysregulated by Aβ might promote significant biochemical alterations to major myelin proteins and directly contribute to white matter pathology frequently seen in AD patients.

## Data Availability

All data generated or analyzed during this study are included in this published article.

## Abbreviations

BCS: Bathocuproine disulfonic acid (selective Cu chelator)
DCKA: 5,7-Dichlorokynurenic acid (glycine site NMDAR antagonist)
NMDAR: N-Methyl-D-Aspartate receptor
NVP: NVP-AAM077 (antagonist of GluN2A-containing NMDARs)
PrP^C^: Cellular prion protein
