# Supplementary material for: Copper ions, prion protein and Aβ modulate Ca levels in central nervous system myelin in an NMDA receptor-dependent manner
Source: Mol Brain. 2022 Jul 26;15:67. doi: 10.1186/s13041-022-00955-2 (PMC9327403; doi:10.1186/s13041-022-00955-2)
Supplement: Supplementary file 1 — Additional file 1. Fig. S1. Freshly dissected optic nerves from mice expressing green YFP in axons, with myelin labeled using the lipid probe Nile Red, were incubated in oxygenated aCSF at 35°C with 1 μM CuSO4 added. Nerves were then fixed in PFA and imaged intact by confocal microscopy. A) After 6 hrs of incubation in aCSF alone, axons and myelin remained morphologically intact. B) In contrast, a 6 hr incubation with addition of the Cu chelator BCS (10 μM) induced significant pathology in the form of axomyelinic spheroids (arrows). C) Quantitative analysis showing mean # of spheroids per 250x250 μm field of view. The BCS-induced pathology was completely blocked by the NMDAR antagonist 5,7-dichlorokynurenic acid (50 μM). [file 13041_2022_955_MOESM1_ESM.pdf]

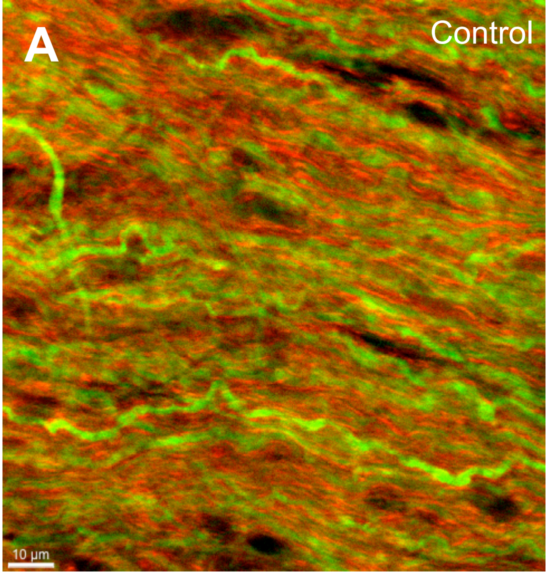

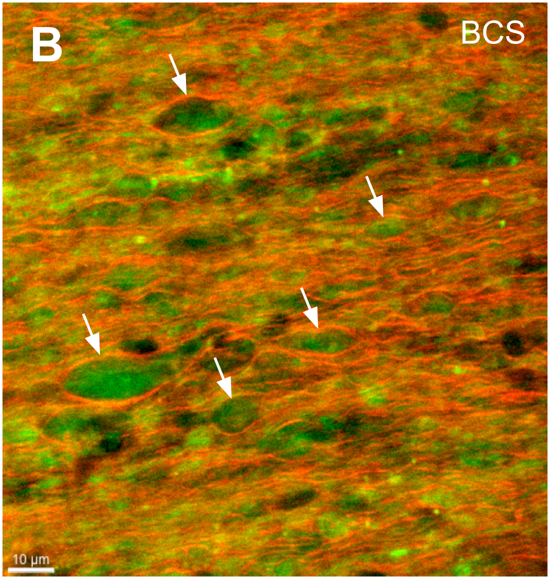

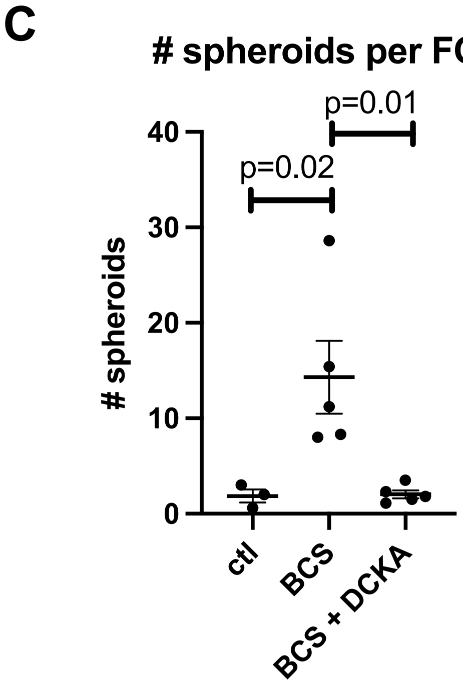

Additional file 1: Freshly dissected optic nerves from mice expressing green YFP in axons, with myelin labeled using the lipid probe Nile Red, were incubated in oxygenated aCSF at 35°C with 1 µM $CuSO_4$ added. Nerves were then fixed in PFA and imaged intact by confocal microscopy. **A)** After 6 hrs of incubation in aCSF alone, axons and myelin remained morphologically intact. **B)** In contrast, a 6 hr incubation with addition of the Cu chelator BCS (10 µM) induced significant pathology in the form of axo-myelinic spheroids (arrows). **C)** Quantitative analysis showing mean # of spheroids per 250x250 µm field of view. The BCS-induced pathology was completely blocked by the NMDAR antagonist 5,7-dichlorokynurenic acid (50 µM).
