# Supplementary material for: Copper ions, prion protein and Aβ modulate Ca levels in central nervous system myelin in an NMDA receptor-dependent manner
Source: Mol Brain. 2022 Jul 26;15:67. doi: 10.1186/s13041-022-00955-2 (PMC9327403; doi:10.1186/s13041-022-00955-2)
Supplement: Supplementary file 2 — Additional file 2. Fig. S2. 9 month old C57 wild type or 5xFAD (transgenic mice harboring 5 human Alzheimer's mutations involving presenilin and APP (Oakley et al., 2006) were immunolabeled for citrullinated myelin basic protein (citMBP, 1B8 antibody) and Aβ (6E10 antibody), and counter-stained with DAPI. Representative micrographs show no amyloid plaque deposition in the wild type mouse as expected and minimal citMBP in the corpus callosum (CC) indicative of healthy myelin. In contrast, the 5xFAD mouse exhibited heavy plaque deposition typically seen at this advanced age, including in the corpus callosum (*). In this white matter tract notable citMBP signal was observed (arrows) consistent with biochemically damaged myelin. These data are consistent with the notion that in vivo Aβ reaches levels sufficient to induce myelin abormalities, which also appears to occur in the human (Additional file 4). [file 13041_2022_955_MOESM2_ESM.pdf]

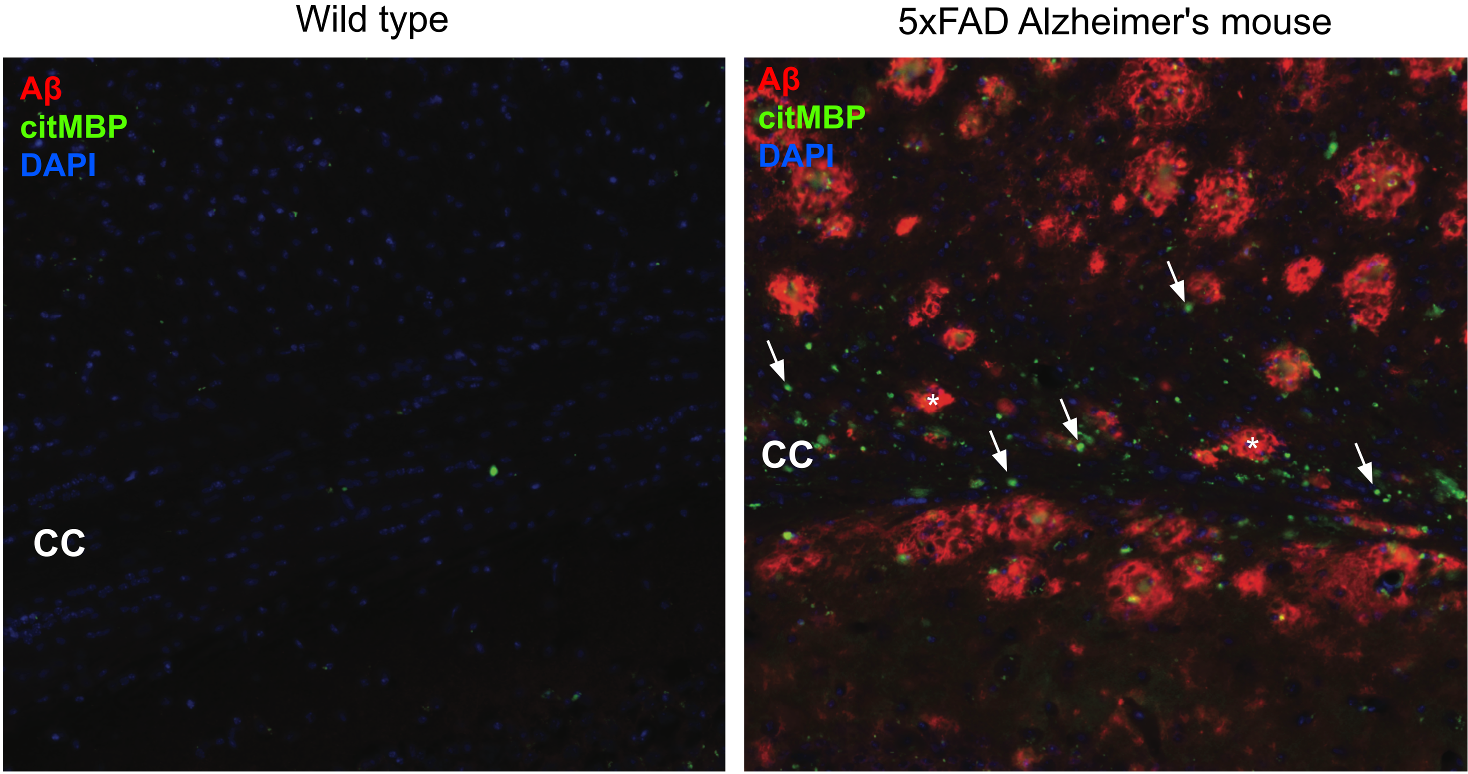

Additional file 2: 9 month old C57 wild type or 5xFAD (transgenic mice harboring 5 human Alzheimer's mutations involving presenilin and APP, Oakley et al., 2006) were immunolabeled for citrullinated myelin basic protein (citMBP, 1B8 antibody) and Aβ (6E10 antibody), and counter-stained with DAPI. Representative micrographs show no amyloid plaque deposition in the wild type mouse as expected and minimal citMBP in the corpus callosum (CC) indicative of healthy myelin. In contrast, the 5xFAD mouse exhibited heavy plaque deposition typically seen at this advanced age, including in the corpus callosum (*). In this white matter tract notable citMBP signal was observed (arrows) consistent with biochemically damaged myelin. These data are consistent with the notion that in vivo Aβ reaches levels sufficient to induce myelin abormalities, which also appears to occur in the human (Additional file 4).
