# Supplementary material for: Copper ions, prion protein and Aβ modulate Ca levels in central nervous system myelin in an NMDA receptor-dependent manner
Source: Mol Brain. 2022 Jul 26;15:67. doi: 10.1186/s13041-022-00955-2 (PMC9327403; doi:10.1186/s13041-022-00955-2)
Supplement: Supplementary file 3 — Additional file 3. Fig. S3. Schematic diagram of the proposed signaling arrangement of myelinic NMDARs and their regulation by Cu ions and PrPc. Action potentials conducted along myelinated axons release glutamate into the periaxonal space (Micu et al, 2016) which, together with the obligatory co-agonist glycine or D-serine, activates myelinic NMDARs resulting in physiological Ca increases in myelin. This is under potent control of Cu ions likely exerting their effects via PrPc ①. Genetic ablation of PrPc increases the sensitivity of NMDARs to agonist leading to increased myelinic Ca entry ②. Acute reduction of Cu levels in the periaxonal space by chelators such as BCS or Aβ1–42 impairs the ability of PrPc to regulate NMDARs resulting in excessive receptor activation and Ca-mediated injury to the myelin sheath. Thus, Cu ions in concert with PrPc may be fundamental regulators of physiological glutamatergic signaling across the axo-myelinic synapse; disruption of this mechanism may represent the earliest steps of demyelinating pathology. [file 13041_2022_955_MOESM3_ESM.pdf]

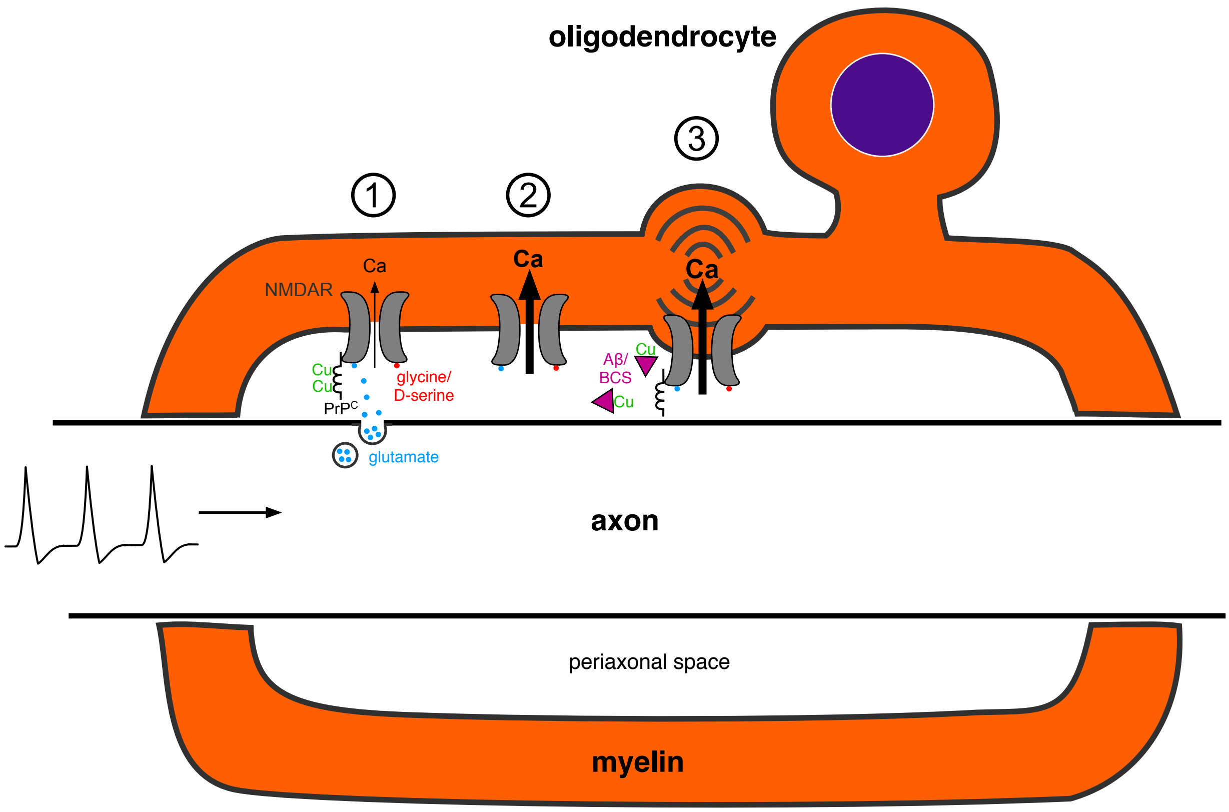

Additional file 3: Schematic diagram of the proposed signaling arrangement of myelinic NMDARs and their regulation by Cu ions and PrP^C. Action potentials conducted along myelinated axons release glutamate into the periaxonal space (Micu et al, 2016) which, together with the obligatory co-agonist glycine or D-serine, activates myelinic NMDARs resulting in physiological Ca increases in myelin. This is under potent control of Cu ions likely exerting their effects via PrP^C ①. Genetic ablation of PrP^C increases the sensitivity of NMDARs to agonist leading to increased myelinic Ca entry ②. Acute reduction of Cu levels in the periaxonal space by chelators such as BCS or $A\beta_{1\text{-}42}$ impairs the ability of PrP^C to regulate NMDARs resulting in excessive receptor activation and Ca-mediated injury to the myelin sheath. Thus, Cu ions in concert with PrP^C may be fundamental regulators of physiological glutamatergic signaling across the axo-myelinic synapse; disruption of this mechanism may represent the earliest steps of demyelinating pathology.
