# Supplementary material for: Copper ions, prion protein and Aβ modulate Ca levels in central nervous system myelin in an NMDA receptor-dependent manner
Source: Mol Brain. 2022 Jul 26;15:67. doi: 10.1186/s13041-022-00955-2 (PMC9327403; doi:10.1186/s13041-022-00955-2)
Supplement: Supplementary file 4 — Additional file 4. Fig. S4. Using standard methods, SDS PAGE of 10% human white matter homogenate from 4 non-neurological control and 3 Alzheimer's disease subjects matched for age. Blots were probed using a proteolipid protein monoclonal antibody (#MA1-80034, Thermofisher). A) In controls, most signal was restricted to the monomeric form, as well as the lower molecular weight DM20 splice variant. In striking contrast, all 3 AD samples exhibited very high molecular weight PLP aggregates that resisted the denaturing conditions of the gel. B) Summary of the densitometry analysis plotted as the integral of very high MW bands > 250 kDa as a ratio of monomer intensity. With the other data presented in the paper, these results are consistent with the notion that excess myelinic Ca accumulation via NMDAR's dysregulated by Aβ might promote significant biochemical alterations to major myelin proteins and directly contribute to white matter pathology frequently seen in AD patients. [file 13041_2022_955_MOESM4_ESM.pdf]

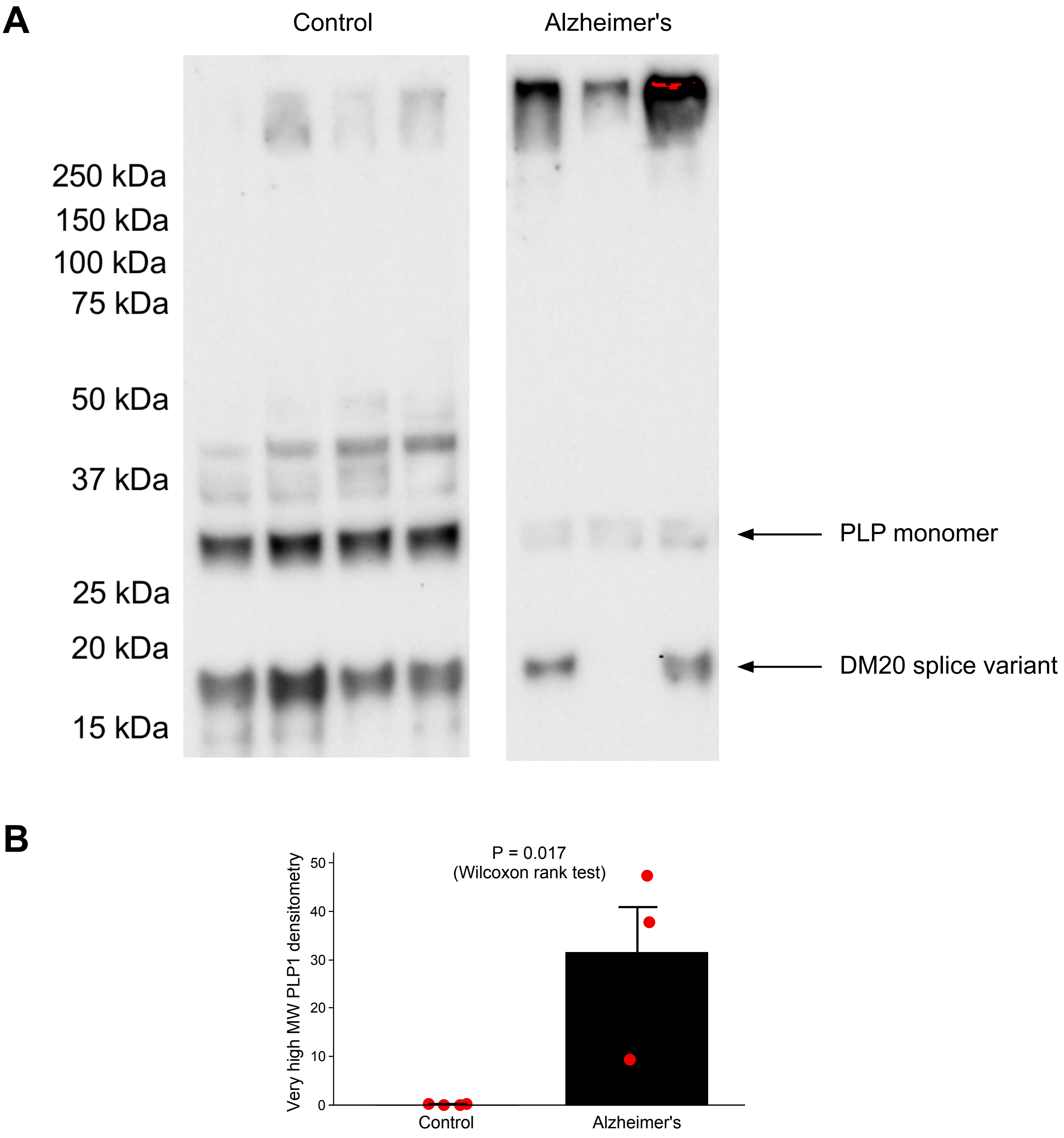

Additional file 4: Using standard methods, SDS PAGE of 10% human white matter homogenate from 4 non-neurological control and 3 Alzheimer's disease subjects matched for age. Blots were probed using a proteolipid protein monoclonal antibody (#MA1-80034, Thermofisher). **A)** In controls, most signal was restricted to the monomeric form, as well as the lower molecular weight DM20 splice variant. In striking contrast, all 3 AD samples exhibited very high molecular weight PLP aggregates that resisted the denaturing conditions of the gel. **B)** Summary of the densitometry analysis plotted as the integral of very high MW bands > 250 kDa as a ratio of monomer intensity. With the other data presented in the paper, these results are consistent with the notion that excess myelinic Ca accumulation via NMDAR's dysregulated by Aβ might promote significant biochemical alterations to major myelin proteins and directly contribute to white matter pathology frequently seen in AD patients.
